# Supplementary material for: Is There Any Improvement of the Coagulation Imbalance in Sickle Cell Disease after Hematopoietic Stem Cell Transplantation?
Source: J Clin Med. 2019 Oct 26;8(11):1796. doi: 10.3390/jcm8111796 (PMC6912463; doi:10.3390/jcm8111796)
Supplement: Supplementary file 1 [file jcm-08-01796-s001.pdf]

Table S1 - Thrombin generation parameters for NC and for SCD children before and after successful allogeneic hematopoietic stem cell transplantation.

Parameters	NC	Before HSCT	3 months after HSCT	6 months after HSCT	9 months after HSCT	12 months after HSCT	15 months after HSCT	Comparison NC versus before HSCT	Comparison before versus 3 months after HSCT
ETP without TM (nM.min)	681.5 (313 – 1355)	1010 (806.6 – 1117)	938.2 (795.3 – 1318)	1021 (803.3 – 1329)	1050 (878.3 – 1261)	925.7 (743.9 – 1298)	992.0 (844.8 – 1263)	P < 0.0001	NS
ETP with TM (nM.min)	426 (157 – 905)	956.1 (753 – 1139)	780.0 (564.7 – 1268)	899.5 (616.4 – 1224)	888.0 (520 – 1103)	768.9 (388.5 – 1189)	870.8 (493 – 1239)	P < 0.0001	NS
Reduction of ETP (%)	61 (34 – 96)	1.4 (-8.4 – 11.2)	12.5 (2.2 – 35.1)	16.2 (3 – 23.3)	21.8 (-1.1 – 40.8)	15.4 (-1.8 – 47.8)	18.3 (1.8 – 41.6)	P < 0.0001	P = 0.0195
Peak without TM (nM)	81.5 (23-173.2)	183.1 (134.7 – 219.3)	136.1 (78.1 – 215.4)	134.9 (64.6 – 208.8)	142.1 (91.2 – 190.1)	129.9 (53.2 – 236.2)	132.9 (106.2 – 192.2)	P < 0.0001	P = 0.0371
Peak with TM (nM)	84 (24.7 – 168)	187.0 (145.9 – 234)	139.6 (78.5 – 225.6)	142.5 (75.3 – 201.9)	151.2 (73.5 – 200.8)	130.0 (47.8 – 243.4)	149.3 (76 – 211.7)	P < 0.0001	P = 0.0371
Velocity without TM (nM/min)	21 (3.8 – 42.7)	62.5 (29 - 78)	31.5 (12 – 52.7)	29 (7.9 – 56.9)	29.8 (18.3 – 67.1)	26.2 (7 – 74.8)	29.8 (20.5 – 66.2)	P < 0.0001	p=0.0098
Velocity with TM (nM/min)	28.9 (6.2 – 68.7)	62.9 (36.5 – 89)	34.4 (15.9 – 64.9)	35 (12.2 – 58.8)	37.8 (17 – 75.1)	30.8 (9.6 – 86.4)	35.6 (18.9 – 63.5)	P < 0.0001	p= 0.0195
Time to peak without TM (min)	9.8 (7.6 – 13)	6.7 (5 – 8.3)	8.1 (6.7 – 11.5)	8.5 (6.4 – 12.3)	8.4 (5.4 – 9.7)	8.7 (7.5 – 11.7)	8.3 (6.4 – 11)	P < 0.0001	p= 0.0137
Time to peak with TM (min)	9.2 (7 – 12.7)	6.6 (5.3 – 8)	7.7 (6.3 – 9.5)	8.2 (6.3 – 10.3)	7.6 (5.3 – 9.5)	7.7 (7 – 10.4)	7.5 (6.3 – 9.7)	P < 0.0001	p= 0.0093
Lagtime without TM (min)	5.3 (3.9 – 8.3)	3.5 (2.6 – 4.0)	3.6 (3.1 – 4.8)	4.0 (3.1 – 4.7)	3.5 (2.6 – 4.5)	4.0 (3.3 – 6.0)	3.7 (2.9 – 6.2)	P < 0.0001	p = 0,0578
Lagtime with TM (min)	6 (4.1 – 9)	3.3 (2.6 – 4.0)	3.7 (3.3 – 4.7)	4.0 (3.1 – 4.5)	3.5 (2.6 – 5)	3.9 (3 – 6)	3.7 (2.9 – 5.8)	P < 0.0001	P = 0,0195

Data are expressed as median and range. ETP: endogenous thrombin potential, TM: thrombomodulin, HSCT: hematopoietic stem cell transplantation.
